# Hospital Notes

**Published:** 1897-01-02

**Authors:** 


### HOSPITAL NOTES.

It is time that the authorities of Guy's Hospital published a precise statement showing exactly how much money was raised at the dinner at which H.R.H. the Prince of Wales presided. We understand that the actual amount received was roughly £166,000 in donations, some of which were promised for a series of years, and upwards of £3,000 in annual subscriptions, which, if capitalised at 3 per cent., would make the total sum resulting from this dinner upwards of £260,000. It is of the first importance that a properly audited and authentic statement should be issued without further delay. We notice that the advertisements are still very inaccurate as to the figures which they contain, and no doubt the new treasurer, Mr. Cosmo Bonsor, now that his attention is directed to the matter, will see that the necessary corrections are made. Financially, Guy's Hospital may be considered to be in a good position, and, if that position is to be maintained and improved, it is essential that figures which are precisely accurate and complete should invariably find their place in the appeals and advertisements which may be issued from time to time.

---

The dinner, at which H.R.H. the Prince of Wales presided, proved more far-reaching in its results than any festival dinner which has ever been held in connection with charity in the history of the world. This result was largely due to the great personal interest and energy which H.R.H. the Prince of Wales exhibited, and it is a strong proof of the powerful influence for good which high personages may exercise in these realms, providing the occasion be adequate to the honour conferred upon any institution by the active association of the heir to the throne with its work and needs. The following letter has been received by the new treasurer, and widely circulated:—

"Marlborough House, Pall Mall, S.W.,

"December, 1896.

"Dear Mr. Cosmo Bonsor,—The generous response by all sections of the public to the appeal which has been made on behalf of the Re-endowment and Sustentation Funds of Guy's Hospital has naturally been a source of great pleasure to the Prince of Wales, and he would be glad if you would add to the official interim report which you are about to issue a paragraph expressing his personal thanks to each donor and subscriber. His Royal Highness, as president of the hospital, cannot, however, but entertain much anxiety concerning its future prospects, and he desires me to remind you that even the considerable measure of financial success which has been already achieved will not, unless further efforts are still made, prove adequate fully to meet the exigencies of the situation.—Believe me, yours truly,

"Francis Knollys.

"H. Cosmo Bonsor, Esq., M.P.,
"Treasurer, Guy's Hospital, S.E."

---

The Hon. Sydney Holland made his first appearance as chairman of the London Hospital on the occasion of the festivities in connection with the Children's Christmas Trees at that institution, which were held on the 28th ult. We congratulate the Hon. Sydney Holland upon the speech which he made in the course of the proceedings. Instead of confusing the issues by giving the outside figures, as has been the case in the past, he told the precise truth, and we have no doubt that the courage thus exhibited in brushing aside the old bad system of exaggeration will produce much larger contributions for the London Hospital than heretofore. Up to quite recently the advertisements have stated that the assured income is only £20,000, and the expenditure £60,000, leaving £40,000 which is required each year from voluntary contributions. No doubt such a statement as this is literally accurate, but it is calculated to mislead the public. Mr. Holland is therefore to be highly commended for amending these figures. His statement, which is the correct statement, was that the expenditure was bound to exceed £60,000 a year, towards which only £46,000 a year was received from invested funds, subscriptions, and donations of all kinds. Legacies were relied on for the balance, but these were so precarious that the committee felt it necessary to beg for further annual subscriptions and donations. It is a great matter to have a chairman of a London hospital with sufficient courage and character to tell the exact truth. If this example be widely followed we make no doubt that the contributions to hospitals will increase everywhere. The public is now alive to the facts, and the statement that a particular hospital requires £40,000 a year beyond its ordinary income when it only needs £16,000 is as ill-advised as it is substantially inaccurate. We hope that many people will testify their approval of Mr. Holland's financial methods by sending a liberal contribution without delay to the London Hospital.

The result of the poll of the trustees of the Manchester Royal Infirmary, which has recently been taken in regard to the question whether to rebuild the infirmary on its present site, in accordance with the plans submitted by the Grand Committee, has resulted in a majority in favour of so doing. The amendment which was proposed was to the effect that it is not desirable to build over more of the ground than is covered by the existing infirmary buildings. It is to be noted that the amendment leaves the field open for action in many directions. The majority in favour of the plan of the Grand Committee was but small, only 46 out of 852 votes polled.

